# An Overview of 10 Years of Activity of a Molecular Laboratory for Buruli Ulcer Diagnosis at a Field Hospital in Benin

**DOI:** 10.1128/jcm.00274-23

**Published:** 2023-05-22

**Authors:** Faraj Fajloun, Line Ganlonon, Ronald Sètondji Gnimavo, Espoir Sodjinou, Akimath Habib, Eric Claco, Irvine Agoundoté, Ambroise Adeye, Perrin Catraye, Charbel Al-Bayssari, Elie Hajj Moussa, Marie Robbe-Saule, Jean Gabin Houezo, Godwin Gérard Kpoton, Adjimon Gilbert Ayélo, Beatriz Gomez, Roch Christian Johnson, Laurent Marsollier, Estelle Marion, Marie Kempf

**Affiliations:** a Universitaire Angers, Nantes Université, CHU Angers, Inserm, INCIT, Angers, France; b Ecole Doctorale en Sciences et Technologie, Université Libanaise, Hadath, Lebanon; c Laboratoire d’Innovation Thérapeutique, Faculté de Sciences 2, Fanar, Lebanon; d Centre de Dépistage et de Traitement de l’Ulcère de Buruli, Fondation Raoul Follereau, Pobè, Benin; e Institut Régional de Santé Publique – Comlan Alfred Quenum, Université d'Abomey Calavi, Ouidah, Bénin; f Department of Medical Laboratory Sciences, Faculty of Health Sciences, University of Balamand, Tripoli, Lebanon; g Programme National de Lutte contre L’Ulcère de Buruli et la Lèpre, Cotonou, Benin; h Fondation Anesvad Bilbao, Bilbao, Spain; i Fondation Raoul Follereau, Health Department, Paris, France; j Département de Biologie des Agents Infectieux, Laboratoire de Bactériologie, Centre Hospitalier Universitaire Angers, Angers, France; University of Manitoba

**Keywords:** Buruli ulcer, PCR, molecular biology laboratory, Benin, *Mycobacterium ulcerans*, diagnosis

## Abstract

Buruli ulcer (BU) is a neglected tropical disease caused by Mycobacterium ulcerans. Early diagnosis is crucial to prevent morbidity. In November 2012, a field laboratory fully equipped for the rapid on-site quantitative PCR (qPCR) diagnosis of M. ulcerans was established at the Buruli ulcer treatment center (CDTLUB) center in Pobè Benin, a region where BU is endemic. We describe its first 10 years of activity and its gradual evolution into an expert laboratory for BU diagnosis. From 2012 to 2022, the laboratory analyzed 3,018 samples from patients attending consultations for suspected BU at the CDTLUB in Pobè. Ziehl-Neelsen staining and qPCR targeting the IS2404 sequence were performed. Since 2019, the laboratory has also received and analyzed 570 samples from other centers. The laboratory confirmed the diagnosis of BU by qPCR for 39.7% samples: M. ulcerans DNA was detected in 34.7% of swabs, 47.2% of all fine needle aspiration samples (FNA) and 44.6% of all skin biopsy specimens. Positive Ziehl-Neelsen staining results were obtained for 19.0% samples. Bacterial load, estimated by qPCR, was significantly greater for the Ziehl-Neelsen-positive samples than for Ziehl-Neelsen-negative samples, and detection rates were highest for FNA samples. Overall, 26.3% of the samples received from other centers were positive for BU. Most of these samples were sent by the CDTLUBs of Lalo, Allada, and Zagnanado, Benin. The establishment of the laboratory in the CDTLUB of Pobè has been a huge success. Optimal patient care depends on the close proximity of a molecular biology structure to BU treatment centers. Finally, FNA should be promoted among caregivers.

**IMPORTANCE** Here, we describe the first 10 years of activity at a field laboratory established at the Buruli ulcer treatment center (CDTLUB) in Pobè, Benin, a country in which Mycobacterium ulcerans is endemic. Between 2012 and 2022, the laboratory analyzed 3,018 samples from patients consulting the CDTLUB of Pobè with a suspected clinical BU. Ziehl-Neelsen staining and qPCR targeting the IS2404 sequence were performed. In total, 39.7% of samples tested positive by qPCR and 19.0% tested positive by Ziehl-Neelsen staining. Detection rates were highest for FNA samples, and the bacterial loads estimated by qPCR were significantly higher for Ziehl-Neelsen-positive samples than for Ziehl-Neelsen-negative samples. Since 2019, the laboratory has also analyzed 570 samples received from outside the CDTLUB of Pobè, 26.3% of which were positive for BU. Most of these samples were sent by the CDTLUBs of Lalo, Allada, and Zagnanado in Benin. The establishment of the laboratory in the CDTLUB of Pobè has been a huge success, with major benefits for both the medical staff and patients. Our findings illustrate that the usefulness and feasibility of having a diagnostic center in rural Africa, where the disease is endemic, is a key part of optimal patient care, and that FNA should be promoted to increase detection rates.

## INTRODUCTION

Neglected tropical diseases (NTDs) are poverty-related and affect almost two billion people, mostly living in tropical and subtropical regions in marginalized communities in Africa, Asia, and the Americas (1). Buruli ulcer (BU) is one such disease. It has cutaneous manifestations and is caused by Mycobacterium ulcerans, a pathogenic environmental mycobacterium that produces a toxin called mycolactone, which has cytotoxic, immunomodulatory, and analgesic properties, leading to severe but painless skin ulcerations (2). Clinically, BU begins with a nodule, papule, edematous lesion, or plaque which frequently progresses to extensive skin ulceration; in the absence of treatment, this can lead to malformations and permanent disability, requiring long periods of hospitalization and surgical interventions that may be prohibitively expensive for poor families. Early diagnosis and treatment initiation are, therefore, essential, to prevent the deleterious consequences of this disease (3, 4).

Two methods are currently used in the field to confirm a clinical diagnosis of BU: Ziehl-Neelsen staining to detect acid-fast bacilli and qPCR for the detection of pathogen-specific DNA (5–8). Quantitative PCR for the IS2404 sequence has proven the most sensitive and specific of the established tests for detecting M. ulcerans, and is therefore currently considered the gold standard for the confirmation of clinical diagnosis (5, 9, 10). The WHO recommends PCR confirmation before the start of treatment to rule out differential diagnoses because this approach can facilitate identification of patients with non-BU chronic ulcers (8). Sampling techniques depend on the nature of the lesions. Fine needle aspiration (FNA) is performed on non-ulcerative lesions (plaques, nodules, and edemas), whereas swabs are taken from under the edges of ulcerative lesions (6, 7). Finally, excised necrotic tissue can be sent to the laboratory, but this sampling method is no longer recommended.

In Africa, BU is common in the central and western regions, particularly Côte d’Ivoire, Ghana, Nigeria, and Benin (11, 12). In 2004, a Buruli ulcer diagnosis and treatment center (Centre de Diagnostic et de Traitement de la Lèpre et de l'Ulcère de Buruli [CDTLUB]) was constructed in Pobè, a city in the Plateau area of Benin known to have a high incidence of M. ulcerans infections. In November 2012, a new laboratory for BU diagnosis by qPCR was established, and its technicians were trained to perform this technique (13). During the first few years, qPCR was systematically performed in duplicate, in the CDTLUB laboratory and the bacteriology laboratory of Angers University Hospital, France, to check the quality of the analysis. The results obtained were good, and, since 2014, the qPCR has been performed at the CDTLUB laboratory in Benin only. Since its creation, this laboratory has participated in different available external quality assessment programs (5, 14). Here, we provide a report of the first 10 years of M. ulcerans diagnostic activities at the CDTLUB laboratory, together with our drawn conclusions and recommendations.

## MATERIALS AND METHODS

Between 2012 and 2022, the CDTLUB laboratory received and analyzed diverse samples consisting of swabs, FNA, and skin biopsy specimens. The laboratory received dried swabs and biopsy specimens or FNA resuspended in water, always transported in an icebox, as recommended by the Standard Operating Protocols of the BU-LabNet (5). Each sample underwent Ziehl-Neelsen staining and qPCR for the IS2404 sequence. Sample preparation and Ziehl-Neelsen staining were performed as previously described (13). Swabs were rehydrated and biopsy specimens were minced in 2 mL of sterile water. From 2012 to 2019, DNA extraction procedures and qPCR were performed as previously described (13). For DNA preparation, 400 μL of sample suspension was centrifuged, and the pellet was resuspended in 50 mM NaOH and heated at 95°C for 10 min. DNA was then isolated using the QIAquick PCR purification kit (Qiagen, Hilden, Germany), in accordance with the manufacturer’s instructions. Negative and positive controls were systematically included. Sample DNA was quantified with an external standard curve constructed with a series of six 10-fold serial dilutions of M. ulcerans (strain 1G897) DNA. Since January 2020, the CDTLUB laboratory of Pobè has been part of the BU-LabNet network and, as such, follows its standard operating procedures for DNA extraction and purification and qPCR. DNA is extracted using the Genolyse kit (Hain LifeScience GmbH, Nehren, Germany), which uses a similar method to the previous one based on an alkaline lysis and heat. Also, quantification is performed using a plasmid instead of purified M. ulcerans DNA (5).

No ethics clearance was required for this retrospective laboratory study.

## RESULTS

### Analysis of samples from patients consulting at the CDTLUB of Pobè.

**FIG 1:**
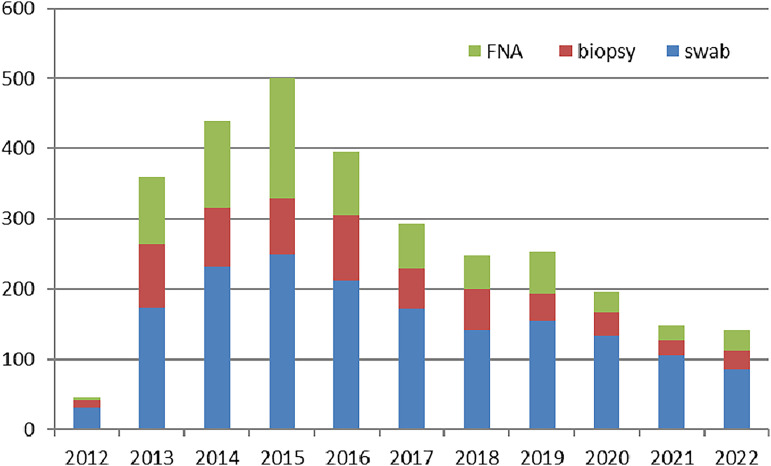
Changes in the numbers of samples from the Buruli ulcer treatment center (CDTLUB) of Pobè, Benin, analyzed by the laboratory between 2012 and 2022 for the detection of Mycobacterium ulcerans.

Between 2012 and 2022, 3,018 samples collected from patients consulting at the CDTLUB of Pobè were analyzed by the laboratory: 1,686 samples (55.9%) were swabs, 742 (24.6%) were FNA samples, and 590 (19.5%) were skin biopsy specimens. The number of analyses performed increased from 2012, reaching 501 samples in 2015. The number of samples analyzed by the laboratory at the CDTLUB of Pobè subsequently decreased to below 200 (Fig. 1). Over the 10 years of activity at this laboratory, swabs were the most frequently tested sample type, accounting for 48.1% (in 2013) to 71.9% (in 2020) of samples. FNA samples accounted for 8.9% of all samples analyzed in 2012, increasing to 34.5% in 2015. Finally, biopsy specimens from excised tissues accounted for 24.4% of analyzed samples in 2012, but only 13.0% in 2021.

**FIG 2:**
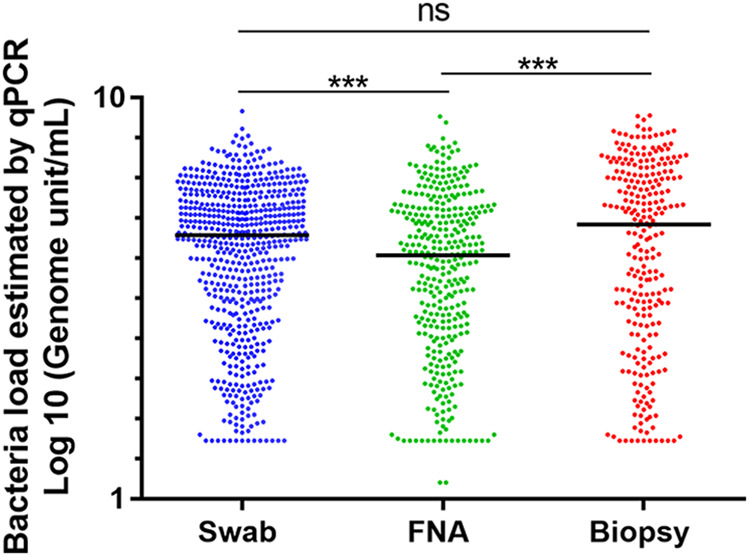
Quantification of M. ulcerans DNA by quantitative PCR (qPCR) analysis on swabs, fine needle aspiration samples, and biopsy specimens (*n *= 3,018). ***, *P* < 0.001; ns, not significant. Bacterial load was assessed by qPCR and is expressed as the number of genome units per mL.

**FIG 3:**
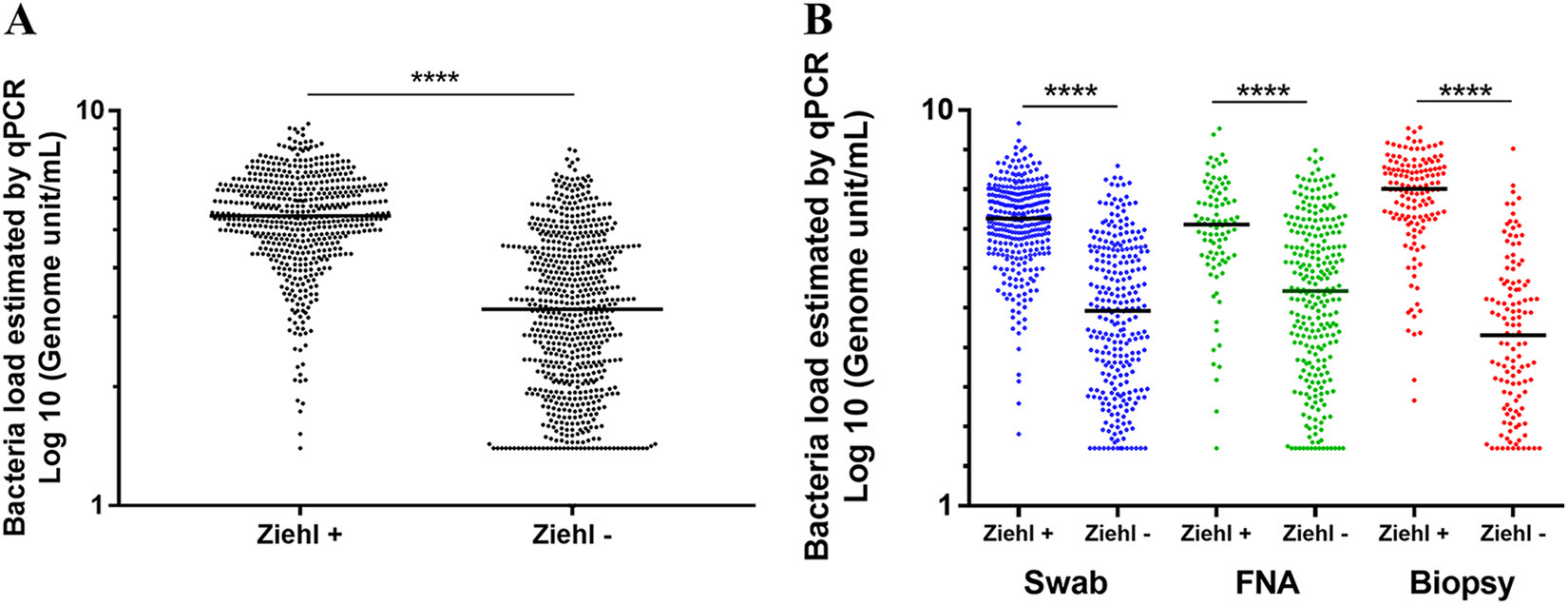
Quantification of M. ulcerans DNA by qPCR by (A) positivity or negativity for Ziehl-Neelsen staining and by (B) sample type and Ziehl-Neelsen staining results. Bacterial load was determined by qPCR and is expressed as the number of genome units per mL. ****, *P* < 0.0001. FNA, fine needle aspiration.

In total, 1,198 (39.7%) samples tested positive for M. ulcerans by qPCR. DNA was detected in 585/1,686 swabs (34.7%), 350/742 FNA samples (47.2%), and 263/590 skin biopsy samples (44.6%) (Table 1, Fig. 2). Ziehl-Neelsen staining was positive for 574 samples (19.0%), with positivity rates highest for biopsy specimens (25.6%), followed by swab samples (19.3%) and then FNA samples (13.2%) (Table 1). Bacterial loads, as estimated by qPCR, differed between sample types. Skin biopsy specimens contained the largest amounts of M. ulcerans DNA, with a mean load of 2.2 × 10^7^ genome units (GU/mL), followed by FNA samples (5.7 × 10^6^ GU/mL) and then swabs (5.4 × 10^6^ GU/mL). The mean load was significantly higher for biopsy specimens than for FNA samples (*P* < 0.001, Cohen’s *d* = 0.0042) and swabs (*P* < 0.001, Cohen’s *d* = 0.20) (Fig. 2). Bacterial load, as estimated by qPCR, was significantly greater for Ziehl-positive than for Ziehl-negative samples (*P* < 0.0001, Cohen’s *d* = 0.23) (Fig. 3A and B).

**TABLE 1:** Comparison between qPCR and Ziehl-Neelsen staining results for the detection of Mycobacterium ulcerans in swabs, FNA samples, and biopsy specimens^*a*^

Sample	Swab	FNA	Biopsy specimen	Total
Total (2012–2022)	1,686	742	590	3,018
qPCR-positive, *n* (%)	585 (34.7)	350 (47.2)	263 (44.6)	1,198 (39.7)
Mean bacterial DNA load estimated by qPCR for positive samples (genome units/mL)	5.4 × 10^6^	5.7 × 10^6^	2.2 × 10^7^	9.2 × 10^6^
Ziehl-Neelsen-positive, *n* (%)	325 (19.3)	98 (13.2)	151 (25.6)	574 (19.0)

a FNA, fine needle aspiration.

### Analysis of samples from patients consulting outside the CDTLUB of Pobè.

From 2019 onwards, the laboratory analyzed a total of 570 samples from patients consulting outside the CDTLUB of Pobè. These samples included 336 from the CDTLUB of Lalo, 123 from the CDTLUB of Zagnanado, and 92 from the CDTLUB of Allada, in Benin (Table 2). These samples accounted for 96.7% of the external samples analyzed. Swabs accounted for 80.4% of samples from Allada, whereas FNA samples accounted for 19.6%. No biopsy specimens were received from Allada. Most (94.9%) of the samples from Lalo were swabs, but 5.1% were FNA samples. The samples from Zagnanado addressed to the CDTLUB of Pobè were either swabs (58.5%) or skin biopsy specimens (41.5%). Positivity rates were highest for the samples from Allada (59.8%), followed by those from Zagnanado (23.6%) and then those from Lalo (19.3%) (Table 2). The other external samples, 13 from Tchaourou Goro, 1 from Cotonou University Hospital, and 1 from Parakou, all tested negative. Finally, the CDTLUB laboratory in Pobè received three samples from Sierra Leone, all of which tested negative, and one sample from Congo-Brazzaville, which tested positive for BU by qPCR (Table 2).

**TABLE 2:** Mycobacterium ulcerans detection results for samples from patients consulting outside the CDTLUB of Pobè^*a*^

Region	Swab		FNA		Biopsy specimen		Total	
	Samples, *n*	qPCR^+^, *n* (%)	Samples, *n*	qPCR^+^, *n* (%)	Samples, *n*	qPCR^+^, *n* (%)	Samples, *n*	qPCR^+^, *n* (%)
Allada	74	46 (62.2)	18	9 (50.0)	0	NA	92	55 (59.8)
Lalo	319	59 (18.5)	17	6 (35.3)	0	NA	336	65 (19.3)
Zagnanado	72	8 (11.1)	0	NA^*b*^	51	21 (41.2)	123	29 (23.6)
Tchaorou Goro	13	0 (0)	0	NA	0	NA	13	0 (0)
CNHU Cotonou	1	0 (0)	0	NA	0	NA	1	0 (0)
PNLUB/Parakou	1	0 (0)	0	NA	0	NA	1	0 (0)
Congo-Brazzaville	1	1 (100)	0	NA	0	NA	1	1 (100)
Sierra Leone	2	0 (0)	1	0 (0)	0	NA	3	0 (0)

a CDTLUB, Buruli ulcer treatment center; qPCR, quantitative PCR; CNHU Cotonou, Cotonou University Hospital; PNLUB, Programme National de Lutte contre L’Ulcère de Buruli; FNA, fine needle aspiration.

b NA, not applicable.

### Time from sample reception to results.

**FIG 4:**
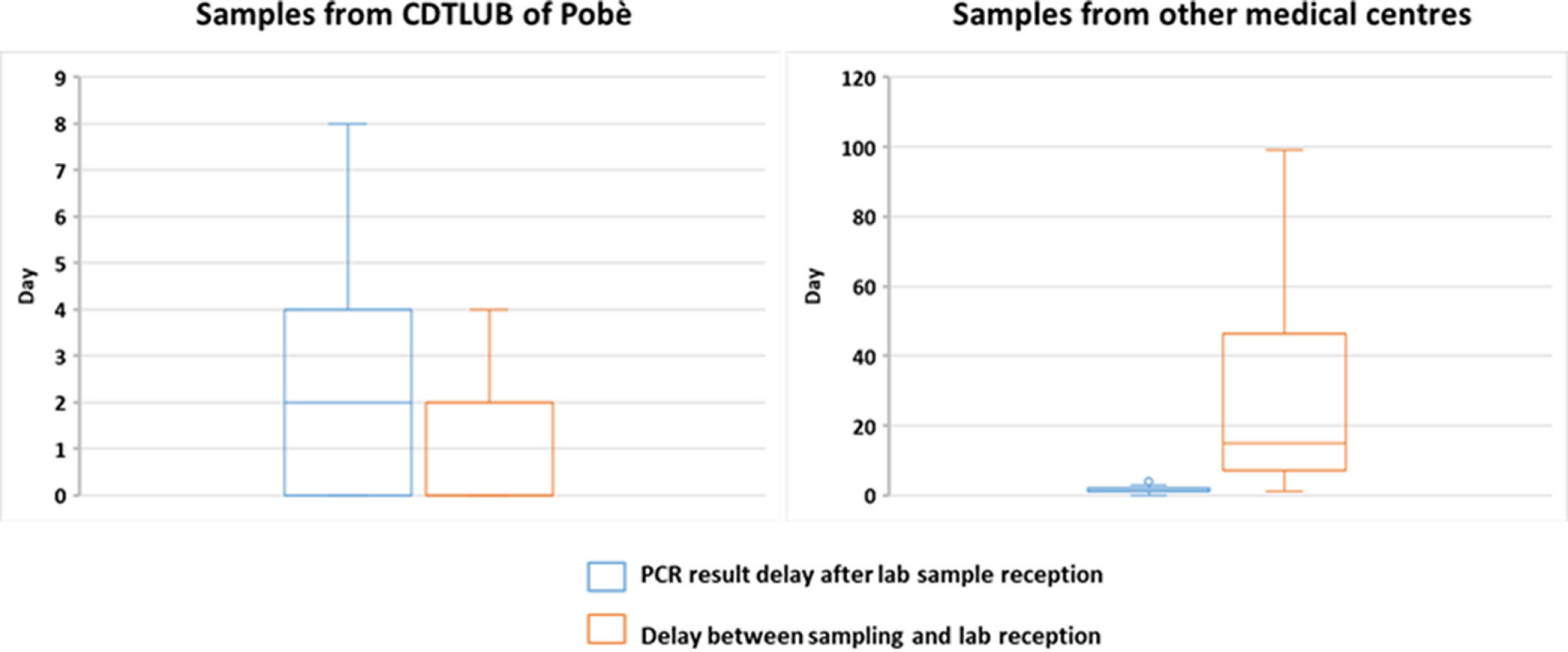
Time to PCR results. Median number of days from sample reception by the laboratory to the delivery of PCR results (blue) and median number of days from patient sampling to reception of the sample by the laboratory (orange). Left: samples from the CDTLUB of Pobè. Right: samples from other CDTLUBs in Benin.

PCR test results must be delivered as rapidly as possible if they are to be useful to clinicians and help improve patient management. We assessed the testing turnaround times using two parameters for the year 2021: time (in days) from reception of the sample at the laboratory to the delivery of PCR results, and time from sample collection from the patient to sample delivery to the laboratory (Fig. 4). We differentiated between samples obtained directly from patients consulting at the CDTLUB of Pobè and samples sent by the other CDTLUBs in Benin. It should be noted that of the 4 CDTLUBs in Benin, only the Pobè center is equipped with a molecular biology laboratory. The median time to PCR results after sample reception was 2 days (interquartile range [IQR]: 0 to 4 days) for samples from the CDTLUB of Pobè and 1 day (IQR: 1 to 2 days) for external samples. As expected, samples were taken and delivered to the laboratory on the same day (median interval: 0 days; IQR: 0 to 2 days) for samples obtained from the CDTLUB of Pobè. However, the time from sample collection to sample delivery to the laboratory was more significant for the samples from the other CDTLUBs (median: 15 days; IQR: 7 to 46.5 days).

## DISCUSSION

Several key findings emerge from our study of the first 10 years of activity at this laboratory. First, our results show that only 19.0% of samples tested positive by Ziehl-Neelsen staining, whereas 39.7% tested positive for M. ulcerans by qPCR. These observations confirm the choice of qPCR as the gold standard for the confirmation of clinical diagnoses of BU. Our findings also highlight the importance of setting up laboratories for rapid and reliable qPCR testing in areas where a disease is endemic, close to the patients. Second, swabs were the most frequently analyzed sample type in our study. However, PCR positivity rates were highest for FNA samples, even though these samples gave lower rates of positivity for Ziehl-Neelsen staining than swabs and skin biopsy specimens. Our findings also confirm the results of our previous studies (6, 13). FNA is generally recommended for closed lesions but can also be performed on ulcerated forms with induration because large numbers of bacilli can be retrieved from these areas. The number of FNA samples analyzed at the CDTLUB of Pobè increased rapidly following the introduction of qPCR testing at this laboratory, but has tended to decrease in recent years. Furthermore, little or no FNA-based sampling is performed at the other CDTLUBs. We can only encourage the more frequent use of FNA instead of, or in addition to, swab sampling. The feasibility of FNA and the availability of consumables for this technique are similar to those for swabbing in Benin. In addition, it has been shown that FNA is less painful than swabbing (6). Given the higher detection rates achieved with FNA, we should therefore encourage the use of FNA for biological sampling in patients with suspected Buruli ulcer.

We noted a decrease in the number of tests performed by the CDTLUB of Pobè from 2015 onwards. These observations may partly account for the decrease in new Buruli ulcer cases in Benin reported since 2012 (15, 16). Since its creation a decade ago, the laboratory of the CDTLUB in Pobè has achieved recognition as a reference laboratory for the diagnosis and analysis of samples from patients with suspected BU. This has led to the center not only performing its own testing autonomously, but also to receiving samples from other CDTLUBs in Benin, with logistic support from the PNLLUB (the national program for Buruli ulcer and leprosy), the Raoul Follereau Foundation, and the Anesvad Foundation. The number of samples received from outside the CDTLUB of Pobè is increasing, probably because the results are obtained very quickly (a median of 1 day after sample reception). Positivity rates were found to be higher for samples from the CDTLUB of Allada than for samples from the other CDTLUBs. The principal reason for this is probably the use of WHO clinical diagnosis scoring criteria by the medical staff of Allada (17), leading to the collection of samples only from patients for whom these criteria indicated the diagnosis to be “very likely BU” or “likely BU.” The other CDTLUBs systematically sample cutaneous ulcers without using this score. Harmonization of the criteria for selecting patients with this scoring grid for biological confirmation is the next objective for the Beninese National Program for Buruli Ulcer (PNLLUB).

Another demonstration of the expertise acquired by the CDTLUB laboratory is its participation as a founding member of the BU-LabNet program, a new model program supervised by the WHO and consisting of a network of 11 laboratories located in African countries where BU is the most endemic, for external quality assessments of PCR-based molecular diagnosis (5). This program supplies all the reagents required for DNA extraction and qPCR amplification free of charge, once annually.

Building on the molecular expertise acquired by the laboratory doctors and technicians following the establishment of qPCR testing for BU diagnosis, an important evolution was observed in 2018 with the implementation of the RLEP-qPCR targeting Mycobacterium leprae DNA specific sequence (18, 19). Currently, during 2023, the aim is to establish GenoType LepraeDR testing (Hain Lifescience GmbH, Nehren, Germany) for the detection of genes conferring resistance to rifampicin, ofloxacin, and dapsone (20); optimize the adaptation of antibiotic treatment; and participate in the surveillance of antimicrobial resistance in leprosy. Finally, following the outbreak of the COVID-19 pandemic in 2020, the CDTLUB laboratory rapidly implemented PCR tests for SARS-CoV-2 at the request of the Health Ministry of Benin. Between April 2020 and August 2021, more than 30,000 PCR tests for SARS-CoV-2 were performed by this laboratory for COVID diagnosis.

### Conclusion.

The establishment of the CDTLUB laboratory in Pobè has been a huge success. The location of this laboratory in a zone in which BU is endemic has been advantageous for both the medical staff and patients. The expertise and dynamism of both the doctors and technicians have enabled the laboratory to invest in other diagnostic missions in addition to that for which it was originally established.
